# TSLP‐targeting therapy: Beyond allergy?

**DOI:** 10.1002/ctm2.1241

**Published:** 2023-05-10

**Authors:** Risa Ebina‐Shibuya, Warren J. Leonard

**Affiliations:** ^1^ Department of Respiratory Medicine Tohoku University Graduate School of Medicine Sendai Japan; ^2^ Laboratory of Molecular Immunology Immunology Center National Heart, Lung, and Blood Institute, National Institutes of Health Bethesda Maryland USA

**Keywords:** tezepelumab, thymic stromal lymphopoietin

## 1 BIOLOGY OF THYMIC STROMAL LYMPHOPOIETIN

Thymic stromal lymphopoietin (TSLP) is a type I four‐alpha‐helical‐bundle cytokine^1^ that is produced by epithelial cells; stromal cells in the lungs, skin and gastrointestinal tract; haematopoietic cells, including dendritic cells (DCs), basophils and mast cells; and hair follicles.^2^ Mechanical injury, TLR2/NOD2, helminth infection, inflammatory cytokines and proteases trigger the production of TSLP by these cells. Pro‐inflammatory cytokines and Th2‐related cytokines also induce TSLP production during inflammatory conditions.^2^ TSLP has multiple functions in both homeostasis and pathological conditions, and this cytokine can act on B cells, DCs, CD4^+^ and CD8^+^ T cells, neutrophils, mast cells, basophils, eosinophils, type 2 innate lymphoid cells, natural killer T cells, smooth muscle cells and even tumour cells.^2^ TSLP shares homology with interleukin (IL)‐7 and signals via a heterodimer of TSLPR (encoded by the *CRLF2* gene) and IL‐7Rα on the surface of target cells, whereas IL‐7 instead signals via IL‐7Rα and the common cytokine receptor γ chain (γ_c_), which shares some similarity with TSLPR,^1^, ^3^ suggesting a historical gene duplication event. TSLP primarily activates STAT5A and STAT5B and more weakly activates STAT1 and STAT3.^4^, ^5^

## 2 TSLP IN ALLERGIC DISEASES

Asthma is a chronic respiratory condition characterised by airway inflammation with eosinophilia and neutrophilia, hyperreactivity and airflow obstruction. Allergen, chemical stimulation and viral infection can each trigger the production of TSLP from epithelial cells, resulting in airway inflammation and airway hyperresponsiveness, leading to the symptoms of asthma. Genetic variations, including TSLP single nucleotide polymorphism rs1837253, result in the onset of asthma, and high levels of TSLP and Th2 cytokines in the lung are observed in the airway in asthma patients. TSLP and IL‐33, a member of the IL‐1 family that drives type 2 responses with increased production of cytokines, including IL‐4, are highly produced in the bronchial epithelium, endothelial cells, mast cells, neutrophils, fibroblasts and submucosa, causing airway obstruction. IL‐4 increases permeability of the airway epithelium by reducing levels of filaggrin and adhesion molecules, and it also increases the expression of IL‐33 and TSLP, leading to the promotion of a Th2 response.

Atopic dermatitis (AD) is a chronic disease characterised by skin inflammation and pruritis. AD is heterogenous with multifactorial pathogenesis, including genetic predisposition as well as environmental and immunological factors. Genetic variants of TSLP affect the severity and persistence of AD, and DNA demethylation of a specific region of the TSLP promoter contributes to TSLP expression in skin lesions of AD patients and perturbed expression of filaggrin. Histamine, a key mediator of allergic diseases, was reported to upregulate TSLP expression in human keratinocytes and nasal epithelial cells by binding to the histamine H4 receptor, suggesting that histamine can promote TSLP‐dependent atopic disease.

## 3 TSLP BEYOND ALLERGY

The role of TSLP in type 2 immune responses in allergic diseases, including asthma, has been extensively studied, and recent studies have revealed additional roles for this cytokine in infectious diseases, cancer, fat metabolism and inflammatory diseases.^2^

During airway infection with influenza virus, pulmonary epithelial cells produce inflammatory cytokines, including TSLP, that alter the immune response in the lungs. In primary infection with influenza virus, there are conflicting reports regarding the role of TSLP on CD8^+^ T cells. One study reported that TSLP does not affect the control of primary influenza infection or viral‐specific CD8^+^ T‐cell responses. Another study showed that TSLP is required for the expansion and activation of virus‐specific effector CD8^+^ T cells in the lung during primary infection but concluded that this was due to an indirect effect based on TSLP‐induced IL‐15 production by CD11b^+^ inflammatory DCs, which in turn acts on CD8^+^ T cells, rather than from direct effects of TSLP on CD8^+^ T cells. Studies using adoptive co‐transfer models of wild‐type and *Crlf2^−/−^* TCR transgenic cells also differed related to the actions of TSLP on CD8^+^ T cells, with TSLP either enhancing primary CD8^+^ T‐cell responses or limiting their responses during primary influenza infection.^6^ These conflicting reports may be due to differences in experimental models (i.e., direct studies in *Crlf2^−/−^* mice vs. co‐transfer models) and virus strains used (i.e., X31 vs. PR8). Recently, TSLP was reported to limit the memory CD8^+^ T‐cell recall response against secondary infection with influenza virus.^6^

Severe acute respiratory syndrome coronavirus 2 (SARS‐CoV‐2), which causes COVID‐19, is associated with cytokine storm characterised by Th1 and Th2 inflammation. Interestingly, TSLP was also induced in COVID‐19 patients, and high TSLP levels correlated with greater severity of COVID‐19. Thus, TSLP might promote this disease, and targeting TSLP is worthy of further evaluation as a possible therapeutic strategy to inhibit Th2 responses in COVID‐19 patients.

TSLP has also been reported to have roles in cancer.^2^ TSLP secretion by cancer‐associated fibroblasts (CAFs) or tumour cells promotes predominantly Th2‐type inflammation in the tumour microenvironment, mostly via DC activation, thereby leading to worse prognosis in pancreatic cancer, breast cancer, gastric cancer and oropharyngeal squamous cell carcinoma. TSLP was secreted by CAFs when activated with tumour‐derived pro‐inflammatory cytokines, including tumour necrosis factor‐α and IL‐1β, and these TSLP‐containing supernatants upregulated expression of TSLPR on myeloid DCs, which secreted Th2‐attracting cytokines and promoted Th2 cell polarisation of CD4^+^ T cells. In contrast, other studies have suggested that TSLP can have tumour‐suppressive activity in breast cancers and cutaneous T‐cell lymphoma.^2^ Thus, TSLP has been associated with both promoting and attenuating the severity of cancer in a range of malignancies, suggesting context‐dependent effects for this cytokine in malignant disease.

## 4 FUTURE OUTLOOK OF TARGETING THERAPY: TEZEPELUMAB

**FIGURE 1:**
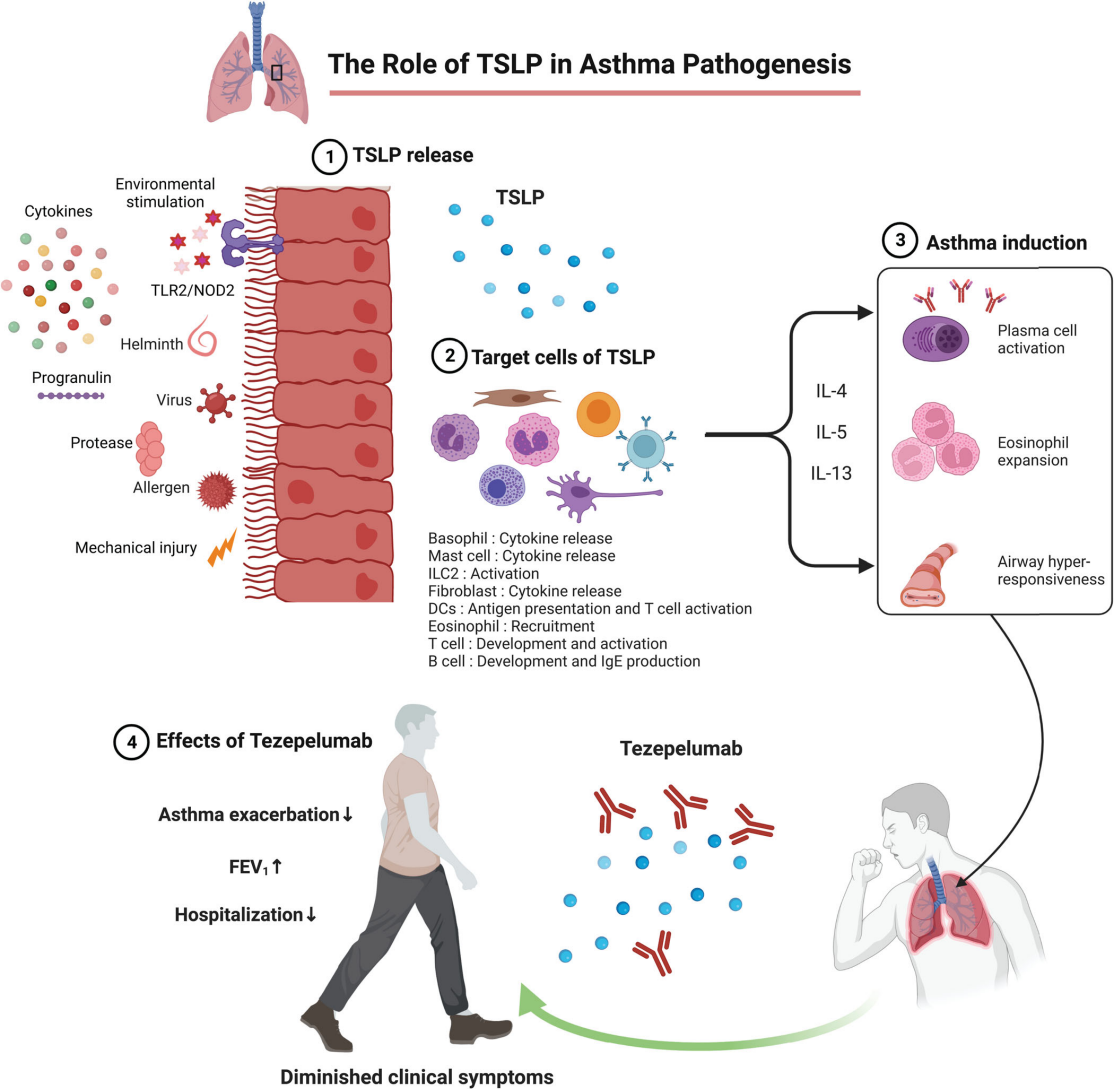
The role of thymic stromal lymphopoietin (TSLP) in asthma pathogenesis. TSLP is released in response to environmental stimulation, cytokines, TLR2/NOD signals, helminth or viral infection, progranulin, proteases, allergens or mechanical injury. TSLP acts on a broad range of target cells, including basophils, mast cells, type 2 innate lymphoid cells (ILC2s), fibroblasts, eosinophils, dendritic cells and B cells. These cells collectively produce interleukin (IL)‐4, IL‐5 and IL‐13, which leads to asthma induction characterised by plasma cell activation, eosinophil expansion and airway hyperresponsiveness. Tezepelumab is a TSLP‐specific antibody that reduces asthma exacerbation and improves prebronchodilator forced expiratory volume in 1 s (FEV_1_), resulting in clinical improvement and a lower rate of hospitalisation. Created with BioRender.com.

A human monoclonal antibody specific for TSLP, known as tezepelumab (Tezspire), was approved by the US Food and Drug Administration in 2021 as an add‐on maintenance treatment for patients aged ≥12 years with severe, uncontrolled asthma. A phase 2 trial (the PATHWAY trial; NCT02054130) showed that tezepelumab reduced clinically significant asthma exacerbations by up to 71% and improved prebronchodilator forced expiratory volume in 1 s (FEV_1_), asthma control and health‐related quality of life compared with placebo.^7^, ^8^ In a phase 3 trial (the NAVIGATOR trial; NCT03347279), patients with severe asthma who received tezepelumab, regardless of the blood eosinophil counts, showed significantly fewer asthma exacerbations, better prebronchodilator FEV_1_, and fewer hospitalisations and emergency room visits compared with the placebo group (Figure 1).^9^ In addition to its approval for asthma, tezepelumab was granted orphan drug designation for the treatment of eosinophilic esophagitis in October 2021 in the USA and is undergoing clinical trials for the treatment of COPD, chronic rhinosinusitis with nasal polyps and chronic spontaneous urticaria. A humanised Fc‐disabled immunoglobulin G1 monoclonal antibody against IL‐7Rα is also being evaluated for treating autoimmune diseases, with the anticipation that it will effectively block the actions of both TSLP and IL‐7.^10^

Given the evidence for multiple functions of TSLP in diseases beyond allergy, it is possible that tezepelumab may have beneficial impact on some of these diseases as well, and further study is warranted regarding targeting TSLP in a broader range of conditions.

## CONFLIFT OF INTEREST STATEMENT

The authors declare no conflicts of interest.

## References

[1] Park LS , Martin U , Garka K , et al. Cloning of the murine thymic stromal lymphopoietin (TSLP) receptor: formation of a functional heteromeric complex requires interleukin 7 receptor. J Exp Med. 2000;192(5):659‐670. doi:10.1084/jem.192.5.659 10974032 PMC2193276

[2] Ebina‐Shibuya R , Leonard WJ . Role of thymic stromal lymphopoietin in allergy and beyond. Nat Rev Immunol. 2023;23(1):24‐37. doi:10.1038/s41577-022-00735-y 35650271 PMC9157039

[3] Pandey A , Ozaki K , Baumann H , et al. Cloning of a receptor subunit required for signaling by thymic stromal lymphopoietin. Nat Immunol. 2000;1(1):59‐64. doi:10.1038/76923 10881176

[4] Rochman Y , Kashyap M , Robinson GW , et al. Thymic stromal lymphopoietin‐mediated STAT5 phosphorylation via kinases JAK1 and JAK2 reveals a key difference from IL‐7‐induced signaling. Proc Natl Acad Sci U S A. 2010;107(45):19455‐19460. doi:10.1073/pnas.1008271107 20974963 PMC2984176

[5] Lu N , Wang YH , Wang YH , Arima K , Hanabuchi S , Liu YJ . TSLP and IL‐7 use two different mechanisms to regulate human CD4+ T cell homeostasis. J Exp Med. 2009;206(10):2111‐2119. doi:10.1084/jem.20090153 19770269 PMC2757885

[6] Ebina‐Shibuya R , West EE , Spolski R , et al. Thymic stromal lymphopoietin limits primary and recall CD8(+) T‐cell anti‐viral responses. Elife. 2021:10. doi:10.7554/eLife.61912 PMC7806261 33439121

[7] Corren J , Parnes JR , Wang L , et al. Tezepelumab in adults with uncontrolled asthma. N Engl J Med. 2017;377(10):936‐946. doi:10.1056/NEJMoa1704064 28877011

[8] Corren J , Gil EG, Griffiths JM , et al. Tezepelumab improves patient‐reported outcomes in patients with severe, uncontrolled asthma in PATHWAY. Ann Allergy Asthma Immunol. 2021;126(2):187‐193. doi:10.1016/j.anai.2020.10.008 33169672

[9] Menzies‐Gow A , Corren J , Bourdin A , et al. Tezepelumab in adults and adolescents with severe, uncontrolled asthma. N Engl J Med. 2021;384(19):1800‐1809. doi:10.1056/NEJMoa2034975 33979488

[10] Ellis J , van Maurik A , Fortunato L , et al. Anti‐IL‐7 receptor alpha monoclonal antibody (GSK2618960) in healthy subjects—a randomized, double‐blind, placebo‐controlled study. Br J Clin Pharmacol. 2019;85(2):304‐315. doi:10.1111/bcp.13748 30161291 PMC6339973

